# Concentration Levels, Biological Enrichment Capacities and Potential Health Risk Assessment of Trace Elements in *Eichhornia crassipes* from Honghu Lake, China

**DOI:** 10.1038/s41598-018-36511-z

**Published:** 2019-02-21

**Authors:** Jingdong Zhang, Yanan Li, Chaoyang Liu, Fei Li, Liyun Zhu, Zhenzhen Qiu, Minsi Xiao, Zhaofei Yang, Ying Cai

**Affiliations:** 1 Research Center for Environment and Health, Zhongnan University of Economics and Law, Wuhan, 430073 People’s Republic of China; 2 School of Information and Safety Engineering, Zhongnan University of Economics and Law, Wuhan, 430073 People’s Republic of China

## Abstract

This study investigated the concentrations of Zn, Cu, Cr, Pb, As and Cd in different tissues of *E*. *crassipes* from Honghu Lake. The total concentrations of trace elements in *E*. *crassipes* were observed in descending order: Zn (111.6162) > Cu (15.7494) > Cr (7.0466) > Pb (5.6251) > As (3.6831) > Cd (0.1941) mg/kg. The order of the bioconcentration factor (BCF) measured in *E*. *crassipes* was Zn > As > Cr > Cu > Pb > Cd > 1, indicating that *E*. *crassipes* possessed a strong biological enrichment ability to accumulate a variety of trace elements. The translocation factor (TF) values decreased in the order of Cu > Zn > Cr > As > Pb > Cd, all of which were lower than 1, which showed that the absorption of the trace elements by *E*. *crassipes* was mainly accomplished in the roots. Moreover, the health risk assessments showed that the carcinogenic and noncarcinogenic risks of the edible parts of *E*. *crassipes* were 26.1 and 4.6 times higher than the maximum acceptable value recommended by the USEPA for adults and children of approximately 39.2- and 6.9-fold, respectively. Children were more sensitive than adults. The main trace elements that led to noncarcinogenic risks were As, Cr and Cu, while Cr and As led to carcinogenic risks. The results of the Pearson correlation showed positive correlations with the concentrations of Zn, Cr and As between *E*. *crassipes* and the water as well as negative correlations of the contents of all six trace elements between *E*. *crassipes* and the sediment.

## Introduction

In recent years, environmental pollution, especially trace elements, has become increasingly severe because of the massive discharge of domestic sewage and industrial waste water. Trace elements, especially metals and metalloids, pose a serious threat to ecosystem and human health because they are nondegradable, have a high toxicity and accumulate easily^1^, and they could be easily enriched by the food chain and threaten food security and eventually cause harm to the human body. Therefore, how to remediate trace element pollution has become an important research goal worldwide^2^. Bioremediation technology is widely applied by most countries due to its low investment and energy consumption compared to physical and chemical treatments^3,4^.

As indicator plants for monitoring trace element pollution in water, *Eichhornia crassipes* (Mart.) Solms (*E*. *crassipes*) have gradually become one of the most important phytoremediations of trace elements because of their great capability to absorb and accumulate trace elements^5^. *E*. *crassipes*, which is a perennial herb in the Pontederiaceae family that originated in South America’s Amazon River, can reproduce quickly^6^. Previous studies have shown that *E*. *crassipes* causes significant effects on many aspects, including socioeconomics, ecology and medicine^5^. The introduction of *E*. *crassipes* brings some benefits but also costs in terms of the treatment of invasive species, with negative effects on navigation, entertainment, and other aspects^7,8^. The solutions to improve biogas yield during the anaerobic digestion of water hyacinth biomass were found to be practical^9^. It is possible to enhance bioethanol production by the combined pretreatment methods for water hyacinth^10^, which could bring some benefits. *E*. *crassipes* can change the turbidity of water and reduce the concentration of dissolved oxygen, nitrogen, phosphorus, ammonia nitrogen, trace elements and other pollutants^11–14^. Many previous studies found that *E*. *crassipes* had a great capacity to absorb and accumulate trace elements^15–17^. *E*. *crassipes* could be used for the removal of chromium (Cr) and zinc (Zn)^18^, and it could also be capable of enriching trace elements such as Fe, Hg^19,20^, As and Pb^21,22^. The ability of different tissues of *E*. *crassipes* to extract and accumulate trace elements is different, and the uptake of trace elements by the roots was reported to be higher than the metal uptake by the shoots^23^. Therefore, *E*. *crassipes* could be used as a phytoremediation plant for the remediation of natural water bodies or wastewater polluted with trace metals^24^. Furthermore, some studies found that *E*. *crassipes* has medical value because it could be used for the treatment of blood disease as well as weight loss, thyroid swelling and other symptoms^25,26^. In addition, the security and health problems associated with *E*. *crassipes* have drawn increasing attention^27^. As was recorded in “*E*. *crassipes* saves the earth”, the tender stems and leaves of *E*. *crassipes* could be eaten, rinsed and made into a soup as well as stir fried or cooled, and the taste was refreshing and delicious^28^. Residents in Taiwan prefer using it as a vegetable salad^29^. The edible aquatic plants bring health risks to human body while serving as a nutritious food^30^. Other aquatic plants such as wild water spinach showed that there were a high health risk caused by trace elements especially for Mn and Cd^31,32^. However, few studies have focused on the health risk assessment caused by the trace elements after *E*. *crassipes* ingestion.

Honghu Lake is the seventh largest freshwater lake in China and is located in the Jianghan Plain between the Yangtze River and its longest tributary, the Han River^33^, and the lake has been listed in the “Ramsar Convention”. Current research has studied water, sediments and fish, indicating that the water quality and the aquatic vegetation in wetlands had obvious spatial distribution characteristics and that plant diversity in the middle and southern areas was obviously higher than that in the north^34^. Due to anthropogenic activities, the water had obvious annual and seasonal changes^35^. In addition, the surface water had been gradually polluted by trace elements, and the trace elements in surface water from Honghu Lake in 2016 were more severely polluted than before^36,37^. The results of the health risk assessment of the trace elements in the surface water from Honghu Lake indicated that the potential carcinogenic risk in each sampling site was approximately at a medium level (10^−5^ to 10^−4^)^36^. For sediment, Cd and Cu were determined to be the pollutants of most concern^38,39^. The fishes captured in the fish studies from Honghu Lake proved to have relatively high accumulated levels of trace elements, and Pb and Cr were recognized as the major health risk contributors for inhabitants through wild and cultured fish consumption^40^. Some local people from Honghu Lake eat water hyacinth. However, few studies concerning the remediation capacity of the aquatic plants in Honghu Lake have examined the health risks caused by *E*. *crassipes* ingestion and the correlations of the trace elements in different environmental media. Thus, it is necessary to evaluate the potential health risk exposure from the trace elements in *E*. *crassipes* from Honghu Lake and to identify the priority pollutants, which might be a new attempt in studies of *E*. *crassipes* from the perspective of environmental health risk.

This study aims to accomplish the following: (1) analyze the absorption status of trace elements such as Zn, Cu, Cr, Pb, As and Cd in the different organs of *E*. *crassipes*, assessing their biological concentration and migration capacities; (2) evaluate the carcinogenic and noncarcinogenic risk levels of the trace elements in the edible parts of *E*. *crassipes* (stems and shoots) from the perspective of health risks; and (3) explore the correlations of the trace elements with the Pearson correlation in water, sediment and *E*. *crassipes*.

## Results

### Physicochemical properties of the water environment in Honghu Lake

The physicochemical properties of the surface water from Honghu Lake roughly reflect the environmental conditions of the effluent. For China’s “surface water environmental quality class II standard”, the results are shown in Table 1. The pH values, dissolved oxygen (DO) levels and electrical conductivity (EC) measures were within the limit range^36^, and the fluctuation in the pH value was not obvious. The average temperature was 27.57 degrees centigrade. The range of turbidity was from 20.7 to 142 NTU, with a mean value of 52.73, which exceeded the national standards for drinking water (3NTU) greatly^41^. Moreover, the average values of total nitrogen (TN) and total phosphorus (TP) exceeded the limit, and the maximum acceptance values of TN and TP were 0.5 and 0.025 mg/L, respectively. As shown in Table 1, the mean concentration of COD was higher than that of the surface water class II standard (15 mg/L), but it reached the grade IV standard of surface water (30 mg/L). Honghu Lake was probably a eutrophic lake at that time.

**Table 1:** The basic physicochemical indexes in surface water samples from Honghu lake.

Parameters	pH	Temperature	Turbidity	DO	EC	TN	TP	COD
		°C	NTU	mg/L	μS/cm	mg/L	mg/L	mg/L
Mean	7.582	27.57	52.73	8.857	273.2	0.54	0.082	27.29
Max	7.79	29	142	11.25	356	0.77	0.19	46.9
Min	7.28	26.4	20.7	6.34	230	0.22	0.04	17.3
SD	0.177	0.716	34.983	1.669	39.534	0.166	0.047	9.441
Chinese standards	6.5–8.5	—	3	6	2000	0.5	0.025	15

A previous study showed that the mean concentrations of Zn, Cu, Cr, Pb, As and Cd in the surface water were 16.057, 3.882, 1.680, 3.561, 0.957, and 0.144 μg/L, respectively. The southern (S4) and northern (S8) areas were seriously contaminated by As, which was considered to be the main pollutant element^36^. With the continuous discharge of domestic sewage, over-reclamation, cultivation, agricultural nonpoint source pollution, etc., the water quality of Honghu Lake declined gradually. Therefore, it is valuable to collect aquatic plants in Honghu Lake that can remedy trace element pollution in the surface water and monitor the water environment.

### Concentration of trace elements in *E. crassipes*

**Figure 1:**
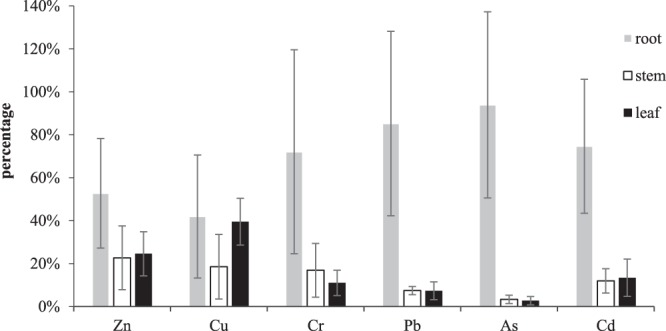
Distribution of trace elements in different tissues samples of *Eichhornia crassipes*.

Phytoremediation is an important method for repairing trace element pollution. This study collected *E*. *crassipes* at 10 different points in Honghu Lake. The accumulation and distribution of different trace elements in the different tissues of *E*. *crassipes* are shown in Table 2 and Fig. 1.

**Table 2:** Concentration of trace elements in different samples of *E*. *crassipes* (mg/kg).

Tissue	Value	Zn	Cu	Cr	Pb	As	Cd
Root	Mean	58.8827	6.6056	5.0783	4.7953	3.4587	0.1449
	SD	±28.4607	±4.5146	±3.3475	±2.4150	±1.5959	±0.0606
	Maximum	98.4064	14.4895	12.1754	8.455	6.9338	0.2411
	Minimum	11.3899	0.0753	0.9428	0.8677	1.6806	0.0639
Stem	Mean	25.3075	2.9205	1.1902	0.4177	0.1234	0.0232
	SD	±16.5549	±2.3709	±0.8818	±0.1073	±0.0697	±0.0110
	Maximum	59.0705	7.584	2.7966	0.625	0.306	0.0384
	Minimum	1.6185	0.3998	0.1003	0.2375	0.0323	0.0093
Leaf	Mean	27.4261	6.2233	0.778	0.4121	0.101	0.026
	SD	±11.4835	±1.7136	±0.4143	±0.2343	±0.0711	±0.0169
	Maximum	47.3645	9.5856	1.9237	0.9662	0.2345	0.0717
	Minimum	9.2231	3.6482	0.5221	0.1128	0.0000	0.0121
Total	Mean	111.6162	15.7494	7.0466	5.6251	3.6831	0.1941
	SD	±39.7700	±7.0125	±3.1963	±2.6340	±1.6688	±0.0722
	Maximum	159.5959	27.7982	14.3834	9.3145	7.0829	0.2993
	Minimum	53.0268	4.9234	3.8478	1.8469	1.8910	0.0968

As shown in Table 2, there were obvious differences in the absorption of various trace elements by *E*. *crassipes*. The total contents of the trace elements Zn > Cu > Cr > Pb > As > Cd declined by 111.6162, 15.7494, 7.0466, 5.6251, 3.6831, and 0.1941 (mg/kg), respectively. The concentrations of Zn, Cu, Cr, Pb, and As were higher than the permissible levels of 20, 10, 0.5, 0.3, and 0.5 (mg/kg), respectively, which were proposed by the Chinese government (GB 2762-2012)^42,43^; this indicates that these concentration might cause a potential risk for humans, while the mean concentration of Cd was within the permissible level of 0.2 mg/kg^42^. In terms of the concentration values, the content of Zn, with a range of 11.39 to 98.41, was the highest and was far higher than the values of the other trace elements, which indicated a significant fluctuation in the Zn concentrations in the *E*. *crassipes* samples from the various sites. According to Table 2, the ability of water hyacinth to absorb and purify Zn and Cu was greater than that for Cr, Pb, As and Cd; thus, *E*. *crassipes* has different abilities to extract and accumulate trace elements. The concentrations of the trace elements in the root decreased in the order of Zn (58.8827) > Cu (6.6056) > Cr (5.0783) > Pb (4.7953) > As (3.4587) > Cd (0.1449) mg/kg, while the contents of Zn, Cu, Cr, Pb, As and Cd in the stems and leaves were 25.3075, 2.9205, 1.1902 0.4177, 0.1234, 0.0232, and 27.4261, 6.2233, 0.7780, 0.4121, 0.1010, and 0.0260 (mg/kg), respectively. In general, the concentrations of the trace elements in the roots were found to be higher than the concentrations in the stems and leaves. Therefore, *E*. *crassipes* could be used for repairing the water environment by Zn and Cu, Cr, Pb and other trace elements, and the deeper mechanism needs to be further researched in the future.

The accumulation capacity of different plant tissues to the same heavy metal, according to Fig. 1, was also quite different. The contents of the trace elements in the roots were generally higher than those in other tissues. In particular, the concentrations rates of the trace elements in the roots were more than 50%, except for Cu (41.94%). The concentrations of Cr, Pb and As in the roots, stems and leaves decreased in turn. The proportions of Cr in the roots, stems and leaves were 72.07%, 16.89% and 11.04%, respectively. More than 85.25% of the Pb was distributed in the roots, and the contents of the stems and leaves were 7.43% and 7.33%, respectively. More than 90% of the As was absorbed by the roots, which was almost 30 times more than that of the stems and leaves. Of all six trace elements, As had the highest relative content of metal in the roots, with less content in the stems and leaves and values of approximately 93.91%, 3.35% and 2.74%, respectively. Zn and Cd were mainly distributed in the roots, and the concentrations of Zn and Cd in the stems and leaves were almost the same. The contents of Zn and Cd in the leaves were slightly higher than those in the stems. For Zn, the proportions in the roots, stems and leaves were 52.57%, 22.67%, and 24.57%, respectively; for Cd, the proportions were 74.66%, 11.93%, and 13.41%, respectively. The contents of Cu in the roots and leaves were high, accounting for 41.94% and 39.51%, respectively.

To better understand the trace elements in *E*. *crassipes* from Honghu Lake, the contents of the selected trace elements were compared with the published data of other aquatic plants at home and abroad, as shown in Table 3. The results illustrated that different aquatic plants had different abilities to enrich trace elements. The concentration of Zn was much higher than that of any other trace elements in these existing studies, except for Nymphoides peltatum. For *E*. *crassipes*, the concentrations of the trace elements decreased in the following order: Zn, Cu > Pb, Cr > As, Cd. The amount of trace elements absorption varies from research sites, which might be related to the degree of pollution of the surrounding environment.

**Table 3:** Comparison of measured trace elements in aquatic plants at home and abroad (mg/kg).

Name of aquatic plants	Zn	Cu	Cr	Pb	As	Cd	Reference
Nymphoides peltatum, China	0.81	0.18	0.206	3.60	1.55	0.10	Sun *et al*.^81^
Reed, China	75.69	37.86	—	10.84	—	1.04	Huang *et al*.^82^
Eichhornia crassipes, China	—	82.25	55.00	25.81	—	0.80	Wang *et al*.^83^
Eichhornia crassipes, China	111.62	15.75	7.05	5.63	3.68	0.19	Present study
Eichhornia crassipes, Malaysia	495.00	402.00	6.20	303.00	0.77	2.40	Kamari *et al*.^25^
V. anagallis-aquatica, Slovenia	69.80	10.10	2.16	1.10	0.37	0.51	Krofli *et al*.^44^
Fontinalis squamosa, Portugal	93.10	20.00	42.70	14.40	10.90	—	Favas *et al*.^45^
Brachythecium rivulare, Portugal	137.00	24.10	53.70	14.80	15.50	—	Favas *et al*.^45^

In general, *E*. *crassipes* had a good accumulation ability to absorb Zn, Cu, Cr, Pb and As, which could be used to repair these trace elements linked with pollution in contaminated water. Especially for the highest content of Zn, *E*. *crassipes* had the strongest accumulation capacity to eliminate Zn. Therefore, compared with the stem and leaf, the root was the tissue that absorbed the most trace elements.

### Bioconcentration factor (BCF) and translocation factor (TF) values for the trace elements in *E. crassipes*

**Figure 2:**
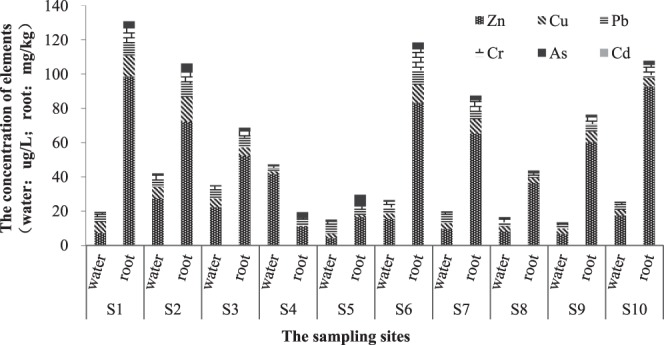
Concentrations of the trace elements in water and root from the sampling sites.

Because trace elements were mainly concentrated in the roots of *E*. *crassipes*, this study used the concentration of the trace elements in the roots to represent the concentration of the trace elements in the plants, and, hereafter, they are the same. The concentrations of the trace elements in *E*. *crassipes* and the surrounding water among the different sampling sites are shown in Fig. 2. The concentration of the trace elements in the different points of different media was quite different, and the contents of the trace elements in *E*. *crassipes* were much higher than those in the surrounding surface water, which indicated that *E*. *crassipes* had a good ability to extract and accumulate trace elements. As shown in Fig. 2, for sites S3 and S2, the trace element concentrations in *E*. *crassipes* gradually increased with the same tendency as those in the water. However, for S1, S6, S7 and S10, the trace element contents in the corresponding water were low, but those in *E*. *crassipes* were relatively high, indicating that *E*. *crassipes* had different abilities to extract and accumulate the trace elements, which is consistent with a previous study^23^.

To better evaluate the ability of *E*. *crassipes* to accumulate the trace elements from Honghu Lake, this study used a bioconcentration factor to measure the absorption capacity of the trace elements by *E*. *crassipes*. As shown in Table 4, Zn had the largest bioconcentration factor, which showed the largest concentration ability of the trace elements. Zn is an essential micronutrient and plays an important role in the synthesis of various proteins and the transformation of various substances in plants^12^. Zn was followed by As and Cr, which were more than 3 times the water concentrations. The contents in *E*. *crassipes* were higher for As than those for Cd only (Table 2), but the bioconcentration factor was lower than that of Zn only, which indicated that As was easy for *E*. *crassipes* to absorb. The concentrations of Cu, Pb and Cd all reached more than 1-fold the concentration in the surrounding water. Therefore, *E*. *crassipes* had different abilities to extract and accumulate the trace elements. The bioconcentration factor of *E*. *crassipes* was observed in the descending order of Zn > As > Cr > Cu > Pb > Cd, all of which were more than 1. In summary, *E*. *crassipes* had a good concentration ability to accumulate a variety of trace elements and could be used for the phytoremediation of Zn, As and Cr and other trace element pollutants in aquatic ecosystems. This study is consistent with reported results^25^, which showed that *E*. *crassipes* had a strong enrichment capacity of trace elements in polluted wetlands and was suitable for water quality biomonitoring and biogeochemical prospecting in fresh water bodies, such as aquatic bryophytes^44,45^.

**Table 4:** BCF and TF values for trace elements in *E*. *crassipes*.

Heavy metal	BCF	TF
Zn	3.667	0.448
Cu	1.702	0.692
Cr	3.023	0.194
Pb	1.347	0.087
As	3.614	0.17
Cd	1.106	0.032

Table 4 lists the transfer factors of the different trace elements in *E*. *crassipes*. As shown in the evaluation results, the transfer factor of *E*. *crassipes* decreased in the order of Cu > Zn > Cr > As > Pb > Cd, which was the same order of the ability to translocate trace elements from the roots to the shoots. All of the values were less than 1, which showed that the uptake of the trace elements by *E*. *crassipes* was mainly concentrated in the roots, which is consistent with previous results^44^.

### Health risk assessment of trace elements in *E. crassipes*

**Figure 3:**
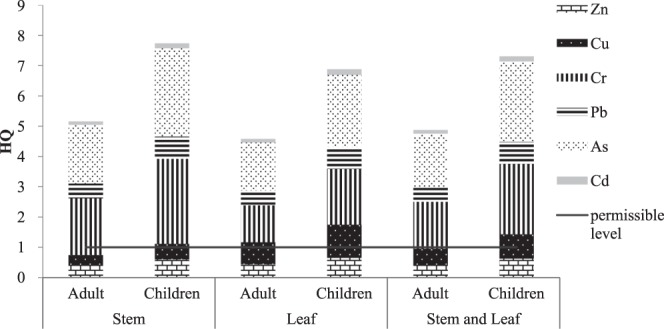
The non-carcinogenic health risk for trace elements caused by *Eichhornia crassipes*.

**Figure 4:**
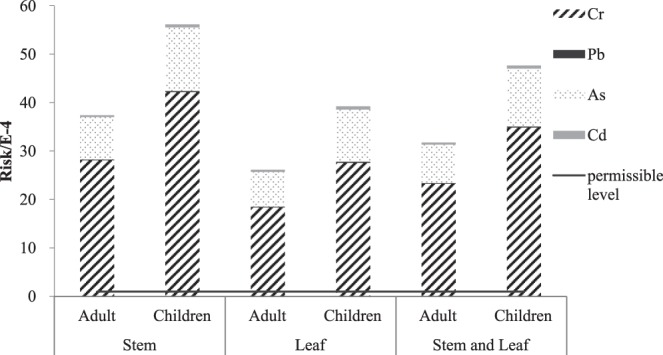
The carcinogenic health risk for trace elements caused by *Eichhornia crassipes*.

*E*. *crassipes* has shown a strong enrichment capacity of several kinds of trace elements; thus, it could be used as a phytoremediation method for water purification. In addition, the young stems and leaves of *E*. *crassipes* tasted clear and refreshing as a vegetable^28,29^, which has an effect of moistening the intestines, and they were also used as vegetable ingredients for the local residents surrounding Honghu Lake. Therefore, it is necessary to study *E*. *crassipes* from the health risk perspective and to preliminarily assess the level of carcinogenic and noncarcinogenic risk to human health after eating the edible portions (stem, leaf, stem and leaf) of *E*. *crassipes*. The results are shown in Figs 3 and 4.

The results showed that the total noncarcinogenic risk (HI) was far higher than the permissible level recommended by the US Environmental Protection Agency^46^. The HI values of children and adults exceeded the permissible level of 6.88-fold and 4.59-fold, respectively. The noncarcinogenic risk level from the edible portions of *E*. *crassipes* was quite obvious. For As and Cr, the level of noncarcinogenic risk in the stems was higher than that in the leaves. For Cu, the leaves had a higher noncarcinogenic risk than the stems. There was little difference in the level of the noncarcinogenic risks of Cd, Pb and Zn from the stems or leaves. The results also showed that children suffered a higher level of noncarcinogenic risk than adults, which indicated that children were more sensitive to trace elements than adults. From the contribution rate of the different trace elements, the noncarcinogenic risk in *E*. *crassipes* from Honghu Lake followed the decreasing order of As > Cr > Cu > Pb > Zn > Cd. As was the highest, with a contribution rate of 34.64% from the leaves, followed by Cr, with a contribution rate of 26.68%, and both were more than 1; almost the same phenomenon was seen in the stems. For Cu, the noncarcinogenic risk on children was greater than 1, while 0.7338 on adults accounted for 16.00%. Therefore, the main trace elements that caused noncarcinogenic risks were As, Cr and Cu.

The total carcinogenic health risk exceeded the permissible levels of 10^−4^ recommended by the US Environmental Protection Agency^46^. Although the trace elements were mainly concentrated in the roots of *E*. *crassipes*, there was a high carcinogenic health risk in the edible portions. The carcinogenic risk followed the decreasing order of stem > leaf and stem > leaf. The total carcinogenic risk levels of the adults and children were 2.61 × 10^−3^ and 3.92 × 10^−3^, respectively, which were far higher than the permissible levels. Although the IR value of the adults was higher than that of the children, the total carcinogenic health risk level was lower than that of the children for each trace element. From the contribution rates of different trace elements, the total carcinogenic risk in the edible portions followed the decreasing order of Cr > As > Cd > Pb. Cr was the highest, accounting for 70.23%, followed by As, accounting for 27.36%. Therefore, the carcinogenic risks caused by Cr and As from *E*. *crassipes* were higher than those of Cd and Pb, which indicated that the main trace elements that caused carcinogenic risks were Cr and As.

According to the results from Figs 3 and 4, *E*. *crassipes* showed a quite obvious health risk to the human body, especially for children, which is consistent with the results of related research reports^47^. Thus, it was not recommended as a common vegetable for the local residents to eat. Regarding the edible portions, both the noncarcinogenic and the carcinogenic risk levels in the stems were higher than those in the leaves. For the typical trace elements, the main pollution elements among the six trace elements of *E*. *crassipes* were As and Cr. Therefore, *E*. *crassipes* consumption should particularly consider the noncarcinogenic and carcinogenic effects of As and Cr. The local residents could control the intake of the trace elements from *E*. *crassipes* by changing their dietary habits. Some relevant departments should pay adequate attention to *E*. *crassipes* intake, especially for children’s health risks as well. Furthermore, other aquatic plants such as mustard showed that there were a high health risk caused by trace elements especially As^48^. For wild water spinach, the health risks needed to be most concerned because the health risk index values were above 1^31,32^. Thus, the edible aquatic plants, as a nutritious food^30^, also brings some health risks to human body, which need to be paid attention by local managers and residents.

### Correlations among trace element concentrations in water, sediments, and *E. crassipes*

A correlation analysis was used to reveal the correlations between the trace element concentrations among the *E*. *crassipes* samples and their corresponding environmental media (water and sediment) by SPSS version 20.0 for Windows. It would be beneficial to explore the correlation of the trace elements and provide evidence for tracking pollution sources. First, the assumption was made that there were no obvious correlations for some kinds of trace elements in the different media. Then, a Pearson correlation analysis was applied to obtain the simple correlation coefficient (P) of the samples with a Kolmogorov-Smirnov test (K-S test) to examine the suitability of the data. If the Pearson’s coefficients were lower than the given value of 0.05 or 0.01, then the null hypothesis was rejected, meaning there was significant linear correlation in the different media; otherwise, if the null hypothesis was accepted, it showed that there was no statistical significance in the different media. The detailed results are shown in Table S1. The data of the trace elements in the sediment are derived from a previous study^49^.

For Zn, a positive correlation (P < 0.01) was found between *E*. *crassipes* and the water (r = 0.704), and a negative correlation (P < 0.01, r = −0.730) was found between *E*. *crassipes* and the sediment. The concentrations of Cr and As in *E*. *crassipes* were (P < 0.01) correlated with those in the water (r = 0.567; r = 0.733) and were negatively correlated with those in the sediments (r = −0.964; r = −0.902). These results showed that the higher contents of Zn, Cr and As in the water led to higher concentrations in *E*. *crassipes* and lower concentration in the sediment. Thus, it may be concluded that the trace elements (Zn, Cr and As) deposited in the sediments from the water were lower than before due to their absorption in *E*. *crassipes*. There were no significant relationships with the concentrations of Cu, Pb and Cd between *E*. *crassipes* and the water, while their concentrations in *E*. *crassipes* were significantly correlated with the sediments (P < 0.01, r = −0.914; P < 0.01, r = −0.971; P < 0.01, r = −0.923). These results indicated that the accumulation of Cu, Pb and Cd in *E*. *crassipes* would reduce their concentrations in the sediments.

Therefore, the higher the contents of Zn, Cr and As in the water were, the higher they were in *E*. *crassipes* and the lower they were in the corresponding sediment. This phenomenon showed that *E*. *crassipes* could absorb elements Zn, Cr and As effectively in water, while the content of trace elements in sediment decreases. In summary, Zn, Cr and As in water migrated from water to *E*. *crassipes* to a lesser extent in sediment. Accordingly, *E*. *crassipes* could be used as a kind of phytoremediation to remedy ecosystems that have been polluted by trace elements. Some relevant could construct and treat the wetlands or introduce the plant into natural water bodies to control the pollution in contaminated water, which is consistent with the results of a previous study^50,51^.

## Discussion

This research showed that *E*. *crassipes* possessed a strong biological enrichment ability to accumulate a variety of trace metals. The absorption of trace elements was mainly concentrated in the roots, which is consistent with the reported results^2,5^. In view of the health risk assessment, the results indicated that there were quite obvious noncarcinogenic and carcinogenic risks to both adults and children, in particular, the total carcinogenic risk values of the adults and children exceeded the permissible level of 26.1-fold and 39.2-fold, respectively. Other aquatic plants such as wild water spinach showed that the HRI (health risk index) value of Cd exceeded the permissible level of 20.18-fold^31^, and the HRI value of As from mustard was 69.86^48^. There were high health risks caused by trace elements^32^. The edible aquatic plants bring health risks to human body while serving as a nutritious food^30^. It’s necessary for some relevant departments to take effective measures for food safety and public health. This study might be the first attempt to assess the health risks from ingesting *E*. *crassipes*. Based on the results of the health risk assessment, we explored the correlation of the trace elements between *E*. *crassipes* and the surrounding environment. The higher the contents of Zn, Cr and As in the water were, the higher the content in *E*. *crassipes* and the lower the content in the corresponding sediment. Interestingly, we found that As and Cr were found to have the highest health risk among the selected trace elements in the surface water from Honghu Lake, as in *E*. *crassipes*^36,52^, while the opposite was observed in the sediment^37,49^.

This study investigated the concentration levels, biological enrichment capacities and potential health risk assessment of the heavy metals in *E*. *crassipes* from Honghu Lake. First, the total contents of the trace elements in the different organs of *E*. *crassipes* decreased in the following sequence: Zn > Cu > Cr > Pb > As > Cd. The bioconcentration factor of six trace elements was higher than 1, while the values of the transfer factor were lower than 1. Second, the health risk assessments showed that the carcinogenic and noncarcinogenic risks of the edible part of *E*. *crassipes* were 26.1 and 4.6 times higher, respectively, than the maximum acceptable value recommended by the USEPA from an adult perspective and approximately 39.2 and 6.9 times higher, respectively, than the value recommended from a child perspective. Third, a Pearson correlation analysis showed that there was a positive correlation among the contents of Zn, Cr and As in *E*. *crassipes* and those in corresponding water as well as a negative correlation with the contents in the corresponding sediment. Furthermore, there are some results worthy of attention. We found that not all sites had the same trend of heavy metal content with the surrounding water, further research could take the plant age and pH into consideration. Based on the results of the health risk assessment, we found that As and Cr were regarded to have the highest risk among the selected trace elements in *E*. *crassipes* from Honghu Lake. Perhaps, further research should assess crowd exposure, such as BW (body weight, kg), IR (daily average intake of pollutants, g/d), and ED (exposure duration to pollutants), etc., which might give more comprehensive results. Overall, this is an exploratory study of *E*. *crassipes* from Honghu Lake, and the results obtained would be useful for further in-depth research into both local and international environmental management in similar areas around the world.

## Materials and Methods

### Study area

Honghu Lake, the seventh largest freshwater lake in China, was listed as an “international important wetland” in 2008 as well as a national nature reserve^36^. Honghu Lake is located in the southern part of Hubei Province and the Jianghan Plain in the southeast, which is in the longitude range from east 113°07′ to 114°05′ and the latitude range from 29°39′ to 30°12′ and lies between the Yangtze River and the East River. The lake area is 348.2 km^2^, 23.4 km from the east to west and 20.8 km from north to south. The average water depth is 1.35 m, ranging from the maximum depth of 2.32 m to the smallest depth of 0.4 m. Honghu Lake is in a subtropical humid monsoon climate, with an average temperature and precipitation of 16.6 °C and 1060.5–1331.1 mm, respectively. In recent years, due to the demand for economic growth in Honghu Lake, the purse seine aquaculture area was almost 103.39 km^2^ ^53^. Meanwhile, a large local tourist company, relying on the abundant aquatic resources of Honghu Lake, was vigorously developing eco-agriculture tourism at the same time as the agriculture industrialization development. The company had an ecological garden of 133.07 km^2^ and a breeding garden of 20.01 km^2^. The area we could actually reach was only approximately 91.73 km^2^. Due to excessive reclamation and cultivation, wanton emissions of domestic sewage and agricultural nonpoint source pollution, the ecosystem was severely damaged^37^. Honghu Lake is a semiclosed lake with relatively low surface water mobility^54^ and has rich animal and plant resources, which is typical in the Jianghan Plain^55^.

### Sample collection and preparation

**Figure 5:**
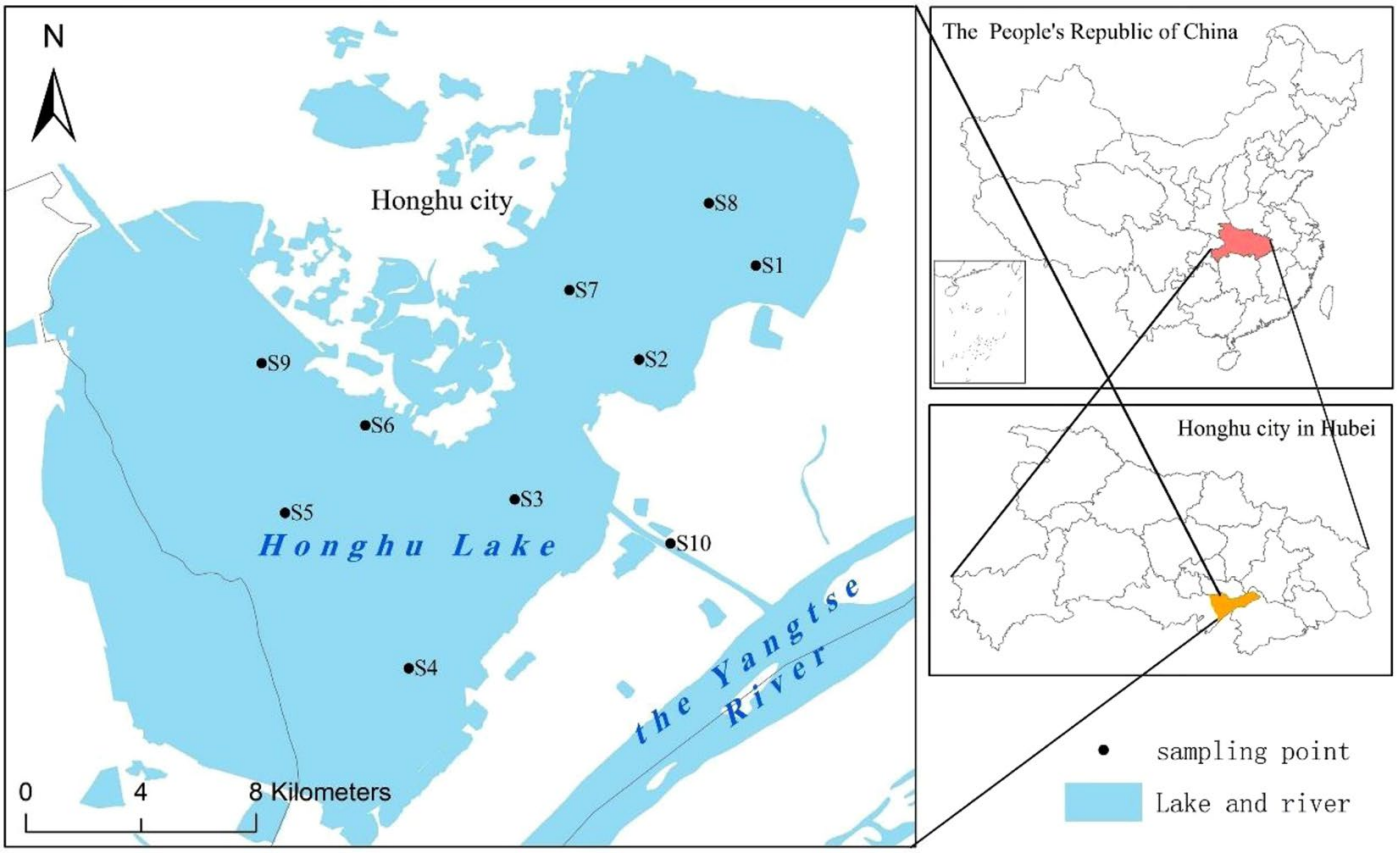
Map of the sampling site locations in Honghu Lake.

Several *E*. *crassipes* samples and the corresponding water samples were collected from 10 different sampling points in Honghu Lake in September 2016. All the *E*. *crassipes* samples were collected by using an underwater sickle, with 50 cm × 50 cm as a sampling unit. The sampling sites were arranged based on the references “Technical Specification for freshwater biological investigation (DB43/T 432–2009)”^56^ and “Determination of Lead, Arsenic, Iron, Calcium, Zinc, Aluminum, Sodium, Magnesium, Boron, Manganese, Copper, Barium, Titanium, Strontium, Tin, Cadmium, Chromium and Vanadium in Foods by Inductively Coupled Plasma Atomic Emission Spectrometry (ICP-AES) DB53/T288–2009”^57^, as shown in Fig. 5, and these sampling points are hereafter referred to as S1-S10. The *E*. *crassipes* samples were pretreated with tap water and then rinsed three times with pure water^11,58^. Subsequently, these samples were separated into three tissues, including roots, stems and leaves, using stainless knives, rinsed with pure water, and then collected into polyethylene containers of equal size. The sampling containers were stored in an oven for thirty minutes at 105 °C and then at 60 °C for twelve to twenty-four hours until the samples dried. The dried samples were ground in ceramic bowls, filtered through 100-mesh sieves and stored in sealed bags of the same size.

The water samples were filtered through 0.45 μm millipore filters and then collected into 1 L polyethylene containers and stored at −4 °C. The pretreatment of the water samples was based on the references “Water Quality–Digestion of Total Metals-Nitric Acid Digestion Method (HJ 677–2013)”^59^ and “Water Quality—Determination of Mercury, Arsenic, Selenium, Bismuth and Antimony–Atomic Fluorescence Spectrometry (HJ 694–2014)”^60^. This paper used a multi-parameter water quality analyzer (HD40Q, HACH, Loveland, CO, USA) to measure the main physicochemical parameters of water, including pH, temperature, dissolved oxygen (DO) and electrical conductivity (EC). Total nitrogen (TN) and total phosphorus (TP) data were measured by the spectrophotometric method referred to in “Determination of total nitrogen in water by alkaline potassium persulfate digestion UV Spectrophotometry (HJ 636–2012)”^61^ and “Determination of total phosphorus in water quality ammonium molybdate spectrophotometric method (GB 11893–89)”^62^.

### Samples digestion and analysis

For the determination of the trace element content in *E*. *crassipes*, a digestion solution was made of 0.2 g *E*. *crassipes* samples blended with 5 ml HNO_3_ and 2 ml H_2_O_2_ and then digested with a microwave digestion instrument. After the acid-driving process on an electric heating plate, the solution was fixed in a 10 ml bottle and stored at −4 °C. After digestion, the total amount of Zn, Cu, Pb, Cr, Cd was detected with atomic absorption spectroscopy (AAS ZEEnit 700P, Jena, Germany), and As was detected by atomic fluorescence spectrometry (AFS-9730, Haiguang Instrument Co. Ltd., Beijing, China). This step was repeated three times. The trace element data in the sediment and the water are derived from previous studies^36,49^. The detection limits of Zn, Cu, Cd, Cr, As and Pb were 0.4, 1, 0.05, 0.1, 0.05 and 0.002 μg/L, respectively.

To ensure the precision and accuracy of the results, quality assurance and quality control were strictly carried out with parallel blank and recovery tests^11,12,63^. Blank tests were performed in every batch of samples processed. The standard curve was drawn when the correlation coefficient was higher than 0.999 for all sample detections. The detection results were reliable when the relative deviations of the parallel sample analyses were controlled within 10% and the accepted recovery rate ranged from 95 to 105%.

### Bioconcentration factor (BCF) and translocation factor (TF)

To evaluate the capacities of *E*. *crassipes* to enrich and transfer trace elements, this study used the parameters bioconcentration factor (BCF) and translocation factor (TF) to analyze the experimental results.

The bioconcentration factor refers to the ratio of the contents of the trace elements in the plants to their surrounding environment (water or sediment)^64^.

1 $$BCF={C}_{i}/{C}_{ei}$$

In this equation, *C*_*i*_ (mg/kg) refers to the residual amount of a certain trace element in the plant. Because the absorption of the trace elements mainly occurred in the roots of *E*. *crassipes*, *C*_*i*_ is content of the trace metals in the roots; *C*_*ei*_ (mg/L) refers to the measured concentration of the trace elements in the environment where the *E*. *crassipes* grew. Because *E*. *crassipes* belongs to the floating plants^65^, *C*_*ei*_ is the content of the trace elements in the surface water in Honghu Lake. The bioconcentration factor is an important parameter for measuring the ability of plants to absorb trace elements^23^. In general, a high BCF value indicates a strong enrichment capacity. In addition, BCF > 1 is an important feature to distinguish the trace element accumulative plants from the common plants^66,67^.

The translocation factor refers to the ratio of the concentration of the trace elements in the shoot of the plants to the roots^68^, as shown in this equation:

2 $${\rm{TF}}={C}_{shoot}/{C}_{root}$$

These two parameters refer to the contents of the trace elements in the shoots and the roots of the plants, respectively. The translocation factor reflects the ability of the trace elements to migrate in plants^69^. A high translocation factor value indicates a strong capacity to migrate, namely, plants could transfer trace elements from the root to the shoot for convenient post-procession^70^.

### Health risk assessment model

Health risk assessment is identified as a model to evaluate the health risks for people who are exposed to harmful factors by estimating the probability of the adverse effects of these factors on the human body^71,72^. Currently, there are three main pathways through which health risks could influence the human body: digestion, dermal absorption and inhalation. The health risk caused by the trace elements in *E*. *crassipes* are mainly due to ingestion into the human body. Our research applies the model RBCA^73,74^ built by the United States Environmental Protection Agency (USEPA) to evaluate the health risks on the human body caused by the six trace elements in *E*. *crassipes*. The exposure dose could be calculated by Equation (3):

3 $$ADD=\frac{C\times IR\times EF\times ED}{BW\times AT}$$

where ADD (mg/(kg·d)) represents the daily average exposure dose of pollutants through digestion, namely, the content of the trace elements absorbed by the human body from *E*. *crassipes* in Honghu Lake in this study; C (mg/kg) represents the concentrations of the pollutants, namely, the exposure concentration of the trace elements in the samples; IR (g/d) is the daily average intake of pollutants, which could be divided into two parts: adults and children, taking the value 301.4 and 231.5 g/d, respectively; EF is the exposure frequency to the pollutants (365 d/a); ED is the exposure duration of the human body to the pollutants (70a); BW represents body weight, taking the value 63.9 and 32.7 kg for adults and children, respectively; and AT(d) is the average exposure time, 25550 d^73,75–78^.

Health risks can be divided into carcinogenic risks and noncarcinogenic risks. Noncarcinogenic risks can be expressed as the ratio of the daily exposure dose to the reference dose, as shown in Equation (4). HQ is the noncarcinogenic risk of a single heavy metal. Neglecting the synergistic and antagonistic effects of the various trace elements, the noncarcinogenic risk of multiple trace element exposure is expressed in HI. As shown in Equation (5), HI is the sum of the HQs. When HQ > 1, noncarcinogenic risk exists.

4 $$HQ=\frac{ADD}{RfD}$$

5 $${\rm{HI}}=\sum _{i}^{n}H{Q}_{i}$$

In these equations, ADD(mg/(kg·d)) is the daily average exposure dose of the pollutants, RfD is the reference dose (mg/(kg·d)), and the reference dose of Zn, Cu, Cr, Pb, As, Cd is 0.3, 4 × 10^−2^, 3 × 10^−3^, 4 × 10^−3^, 3 × 10^−4^ and 1 × 10^−3^, respectively^75,76,78,79^. HQi is the noncarcinogenic risk caused by some sort of trace elements through digestion, and i, n represents the element species.

Carcinogenic risks can be evaluated by Equations (6) and (7). Equation (6) indicates the single carcinogenic risk of the pollutants, ignoring the synergistic and antagonistic effects of the various trace elements, while the compound carcinogenic risk is expressed by Risk_T_. Risk_T_ is the sum of Risk_i_. The USEPA believes that the maximum acceptable risk of cancer in the human body is 1 × 10^−4^ ^46^. When Risk_T_ > 1, carcinogenic risk could be considered.

6 $$Ris{k}_{i}=ADD\times SF$$

7 $$Ris{k}_{T}=\sum _{i}^{n}Ris{k}_{i}$$

As shown in Equations (6) and (7), ADD (mg/(kg·d)) is the daily average exposure dose of the pollutants, SF represents the carcinogenic slope factor (kg·d/mg), and SF(Cr) = 0.5, SF(Pb) = 0.0085, SF(As) = 1.5, and SF(Cd) = 0.38^46,76,77,79^. Because Zn and Cu are essential trace elements for plants and are not pollutants according to the national standard of food safety in China (GB 18406.1-2001)^80^, the carcinogenic risks of Zn and Cu are not considered.

### Statistical data analysis

A Pearson correlation was used to analyze the correlation between the trace element concentrations in *E*. *crassipes* and their corresponding environment (water and sediment) to explore whether trace elements have a certain pathway of migration. All samples were analyzed in SPSS 20.0, including the Kolmogorov-Smirnov test (K-S test), to check for normality with a significance level of 0.05 or 0.1.

## Electronic supplementary material

Supplementary Information
