# Supplementary material for: Concentration Levels, Biological Enrichment Capacities and Potential Health Risk Assessment of Trace Elements in Eichhornia crassipes from Honghu Lake, China
Source: Sci Rep. 2019 Feb 21;9:2431. doi: 10.1038/s41598-018-36511-z (PMC6385497; doi:10.1038/s41598-018-36511-z)
Supplement: Supplementary file 1 — Supplementary Information [file 41598_2018_36511_MOESM1_ESM.pdf]

Concentration Levels, Biological Enrichment Capacities and Potential Health Risks Assessment of Trace elements in *Eichhornia crassipes* from Honghu Lake, China

Jingdong Zhang[1,2], Yanan Li[1,2], Chaoyang Liu[1,2] *, Fei Li[1,2], Liyun Zhu[1,2], Zhenzhen Qiu[1,2], Minsi Xiao[1,2], Zhaofei Yang[1,2], Ying Cai[1,2]

Supporting information

Table S1. Correlations among trace element concentrations in water, sediments and *Eichhornia crassipes*

**Zn**	**Water**	**Sediment**	***Eichhornia crassipes***
Water	1.0000		
Sediment		1.0000	
Eichhornia crassipes	0.704 **	-0.730**	1.0000

**Cu**	**Water**	**Sediment**	***Eichhornia crassipes***
Water	1.0000		
Sediment		1.0000	
Eichhornia crassipes	0.3740	-0.914**	1.0000

Cr	Water	Sediment	***Eichhornia crassipes***
Water	1.0000		
Sediment		1.0000	
Eichhornia crassipes	0.567**	-0.964**	1.0000

Pb	Water	Sediment	***Eichhornia crassipes***
Water	1.0000		
Sediment		1.0000	
Eichhornia crassipes	0.3040	-0.971**	1.0000

As	Water	Sediment	***Eichhornia crassipes***
Water	1.0000		
Sediment		1.0000	
Eichhornia crassipes	0.733**	-0.902**	1.0000

Cd	Water	Sediment	***Eichhornia crassipes***
Water	1.0000		
Sediment		1.0000	

Eichhornia crassipes	0.0260	-0.917**	1.0000

**：significant correlation at the 0.01 level (bilateral)

Table S2. The record of Eichhornia crassipes sampling sites

2016.09.17 sunny 29.5℃ recorder：Ying Cai		
Site	photo number	time
S1	S1-1, S1-2	10:47
S2	S2-1, S2-2, S2-3	10:00
S3	S3-1	9:03
S4	S4-1, S4-2, S4-3	15:02
S5	S5-1	14:36
S6	S6-1, S6-2	13:31
S7	S7-1, S7-2, S7-3	11:57
S8	S8-1, S8-2, S8-3	11:21
S9	S9-1	14:00
S10	S10-1, S10-2	8:15

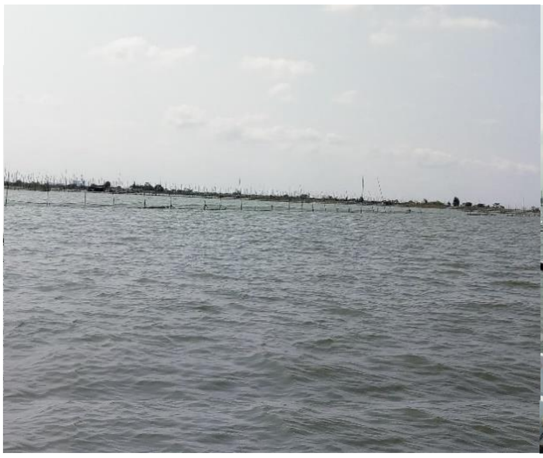

S1-1

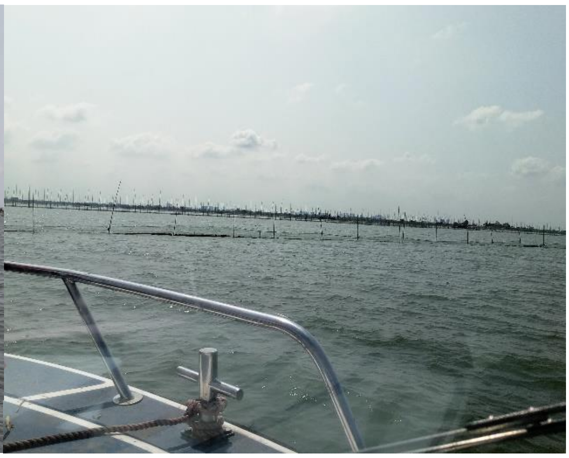

S1-2

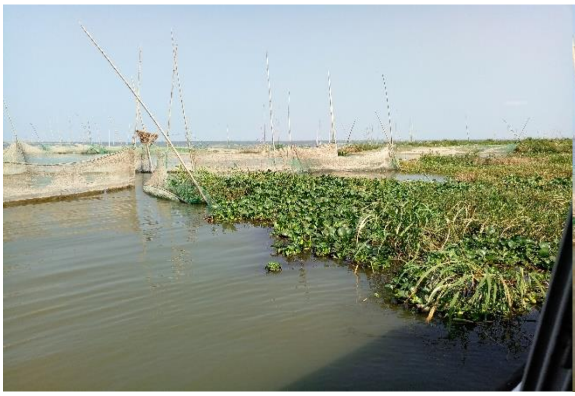

S2-1

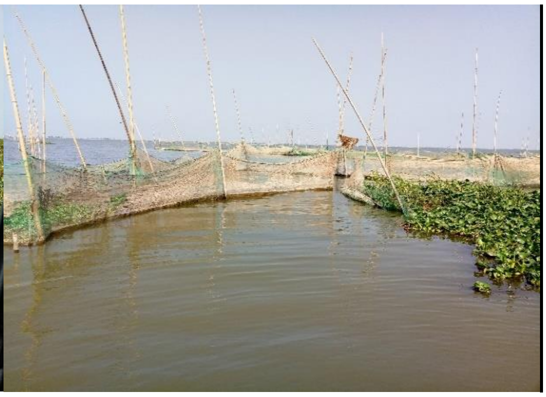

S2-2

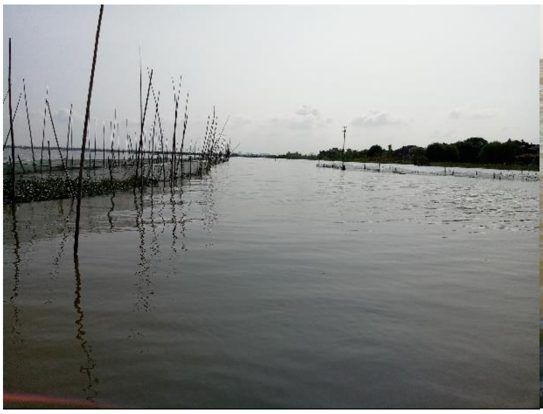

S2-3

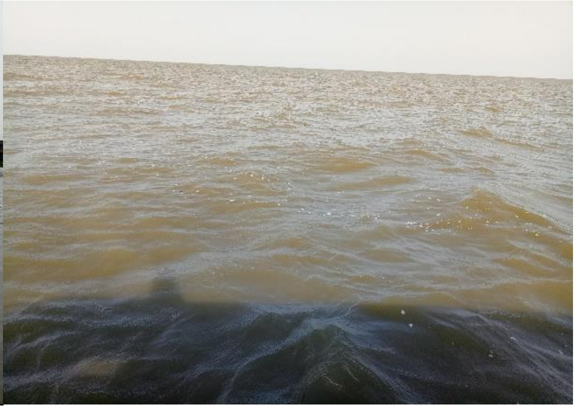

S3-1

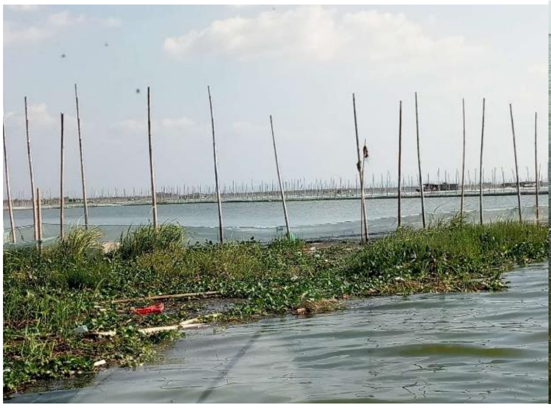

S4-1

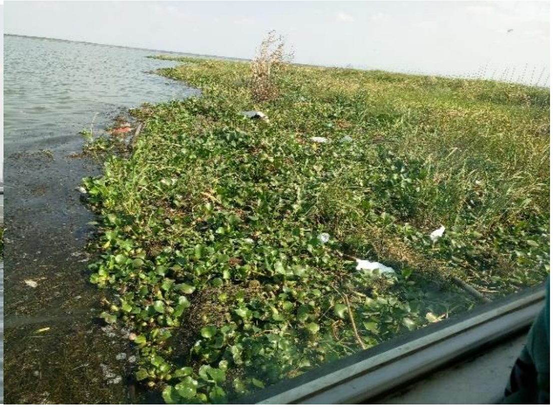

S4-2

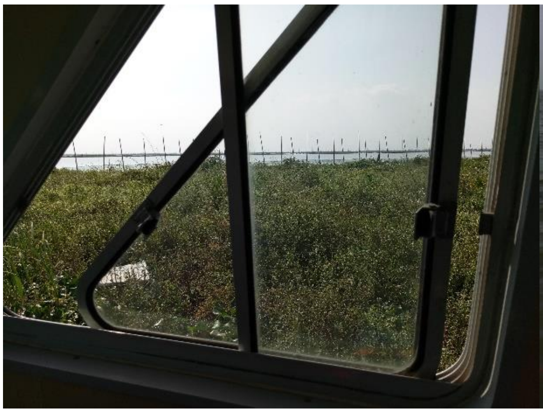

S4-3

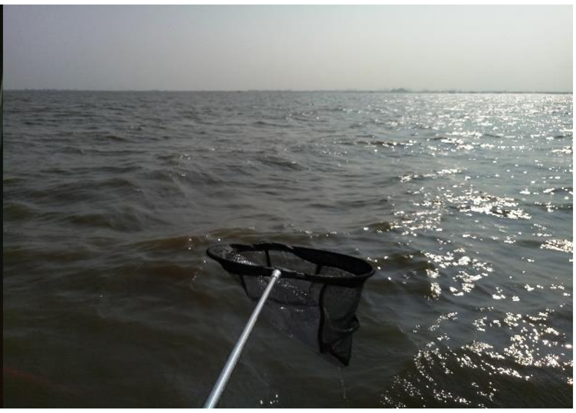

S5-1

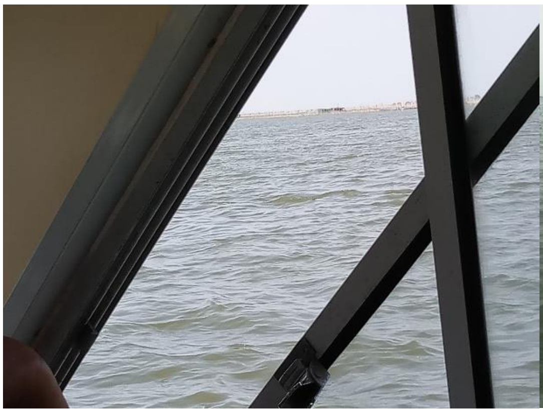

S6-1

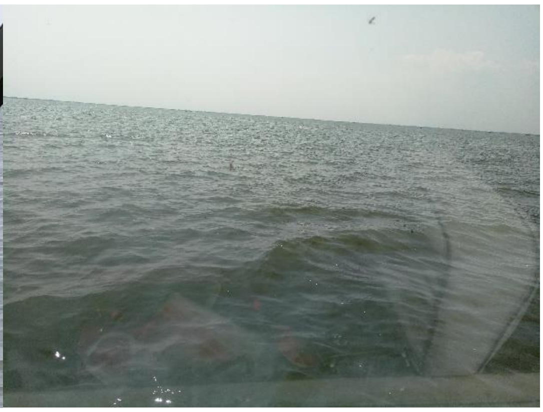

S6-2

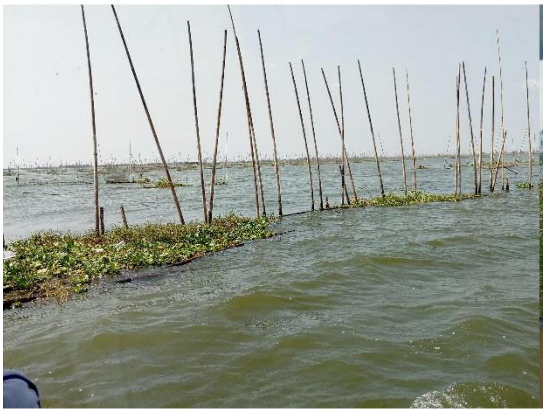

S7-1

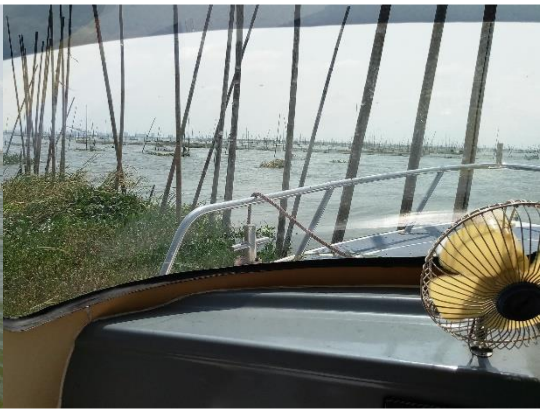

S7-2

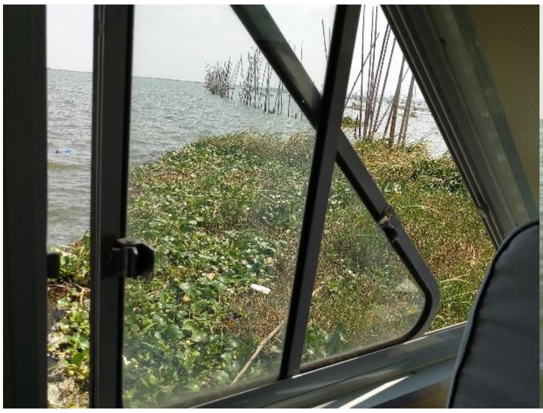

S7-3

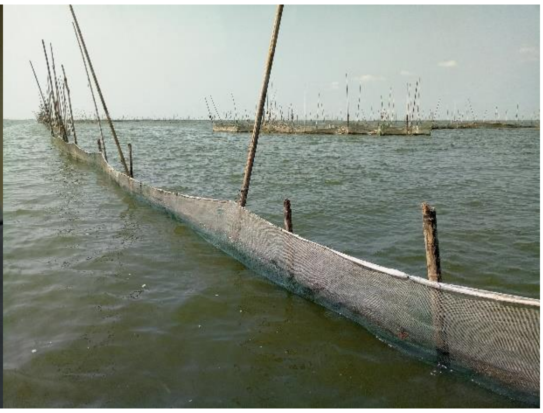

S8-1

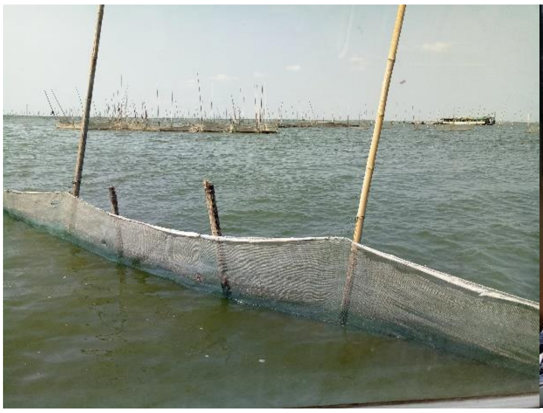

S8-2

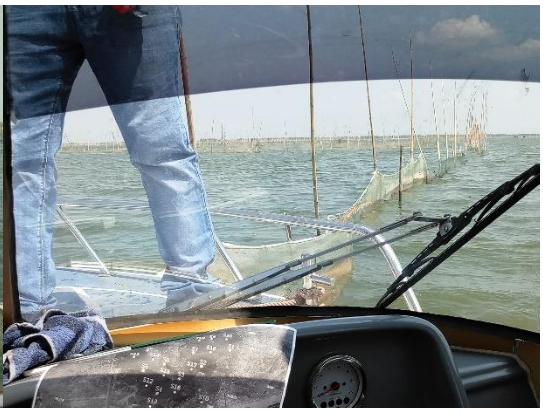

S8-3

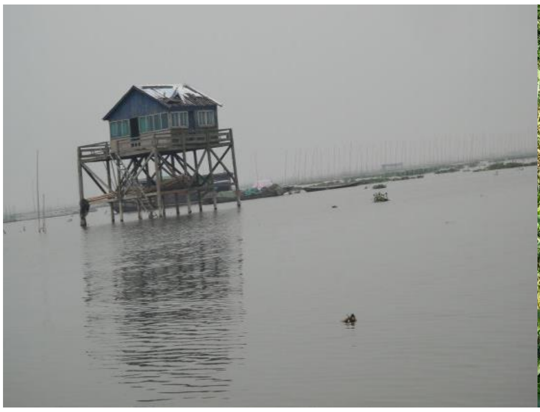

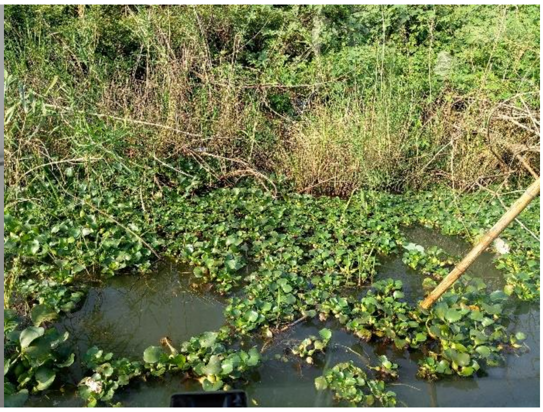

S9-1 S10-1

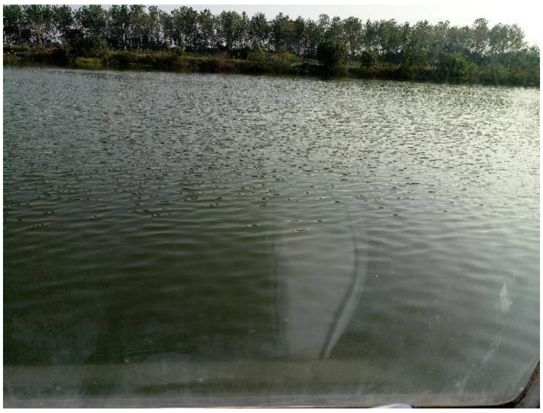

S10-2

Figure S1. The developing purse seine aquaculture in Honghu Lake at each sampling site

Table S3. The water quality data for the various sampling sites

Parameters	pH	Temperature	Turbidity	DO	EC	TN	TP	COD
		℃	NTU	mg/L	μS/cm	mg/L	mg/L	mg/L
S1	7.7	27.1	44.3	8.41	260	0.56	0.06	22.2
S2	7.43	26.8	94.6	6.84	269	0.61	0.13	20.7
S3	7.57	26.4	142	6.34	276	0.65	0.19	22.7
S4	7.67	29	34.4	10.65	232	0.77	0.04	46.9
S5	7.68	28	39.9	11.25	230	0.74	0.04	44.1
S6	7.64	27.9	37	10.57	338	0.59	0.1	17.3
S7	7.29	27.2	42.1	8.81	259	0.31	0.04	25.2
S8	7.79	27.3	34.1	9.54	259	0.47	0.06	26.6
S9	7.28	28.1	38.2	9.44	356	0.22	0.11	24.6
S10	7.77	27.9	20.7	6.72	253	0.48	0.05	22.6
